# Translational prospectives for deep brain stimulation and low-intensity focused ultrasound neuromodulation: IFCN Handbook chapter

**DOI:** 10.1016/j.cnp.2026.03.008

**Published:** 2026-04-10

**Authors:** Wolf-Julian Neumann, Ghazaleh Darmani

**Affiliations:** a Movement Disorder and Neuromodulation Unit, Department of Neurology, Charité—Universitätsmedizin, Berlin, Germany; b Division of Neurosurgery, Department of Surgery, University of Toronto, Toronto, Canada; c Krembil Research Institute, University Health Network, Toronto, Canada

**Keywords:** Deep brain stimulation, Transcranial ultrasound stimulation, Neuromodulation, Neurological disorders, Psychiatric disorders

## Abstract

The translation of experimental brain stimulation procedures to clinical practice remains a significant challenge. In the present book chapter we review emergent innovations that have recently surfaced or are imminent to make the leap, improving the treatment of patients with brain disorders. We focus on two domains of accelerated innovative potential in both invasive and non-invasive brain stimulation domains. First, current advances in deep brain stimulation (DBS) methodology are introduced. DBS is an established treatment for neurological disorders such as Parkinson’s disease, essential tremor and dystonia. Emerging technological advances focusing on when and how to stimulate allow for an extension and refinement of DBS promising scientific and clinical breakthroughs. We reflect on the unprecedented opportunities that MRI-based connectomics and neurophysiology based closed-loop DBS provide for patients with movement disorders, epilepsy and neuropsychiatric diseases. Next, we explore low-intensity focused transcranial ultrasound stimulation (TUS) as an emergent non-invasive neuromodulation technique. Unlike electrical or magnetic methods, TUS combines millimeter spatial precision with the ability to reach deep brain targets, making it the closest non-invasive analogue to DBS. We summarize its candidate mechanisms of action, highlight advances in transducer hardware and skull aberration-correction strategies, and review early clinical studies across movement disorders, epilepsy, pain, and psychiatric indications. Together, these developments illustrate how TUS is progressing from proof-of-concept experiments toward biomarker-guided therapeutic protocols. In conclusion, this chapter provides an outlook on the future of invasive and non-invasive techniques, emphasizing the need for continued research and innovation to overcome challenges in translating experimental successes into effective clinical therapies.

## Highlights

• Connectomic and adaptive deep brain stimulation enable circuit-guided therapies.
• Focused ultrasound is the closest non-invasive analogue to deep brain stimulation.
• Invasive and non-invasive approaches converge toward personalized neuromodulation.

## 1 Introduction

The rapid advancement of neurotechnological innovations has introduced a new era in therapeutic clinical neurophysiology, offering promising tools to enhance patient outcomes. However, the journey from experimental discovery to clinical application remains a significant challenge, due to safety concerns, regulatory hurdles, and the need for robust evidence of efficacy. Among these advancements, both non-invasive and invasive brain stimulation techniques have garnered attention. Methods such as low-intensity focused transcranial ultrasound stimulation (TUS) and deep brain stimulation (DBS) have shown remarkable potential for using technological innovation to improve the treatment of neurological and psychiatric disorders (Neumann et al., 2023b). Yet, the integration of these technologies into routine clinical practice requires a careful balance of innovation and caution (Neumann et al., 2023a). In this review, we will explore the latest developments in brain stimulation techniques and discuss the major strides made towards clinical adoption. We will also provide an outlook on how these emergent techniques are being translated into clinical practice, highlighting the opportunities and challenges that lie ahead in the evolving landscape of therapeutic clinical neurophysiology. This discussion aims to inform and guide clinicians, researchers, and policymakers as they navigate the complexities of implementing these cutting-edge technologies to improve patient care. We first introduce the state-of-the-art in DBS, before describing the pathway for deep brain neuromodulation with transcranial ultrasound stimulation as a non invasive alternative.

## 2 Emergent innovations in invasive deep brain stimulation

### 2.1 Principles and mechanisms

#### 2.1.1 Local DBS effects at synaptic and neuronal population level

Deep brain stimulation (DBS) is a well-established therapy for movement disorders such as Parkinson’s disease and dystonia. It is also applied under humanitarian exemption for obsessive–compulsive disorder and continues to be investigated in conditions such as depression, epilepsy and Alzheimer’s disease (Neumann et al., 2023a, Neumann et al., 2023b). Initially, DBS was conceptualized as a focal therapy: electrodes were implanted into subcortical nuclei such as the subthalamic nucleus (STN), globus pallidus internus (GPi) or thalamus with the assumption that local inhibition or excitation would directly relieve symptoms. Subsequent experimental and clinical work, however, has revealed that the mechanisms are considerably more complex. At a physiological level, DBS delivers high-frequency electrical pulses (typically 130–180 Hz, 30–200 μs pulse width, 1–6 V or 0.5–5 mA amplitude) that activate axons in the vicinity of the electrode, producing both local and distant effects through orthodromic and antidromic conduction as well as collateral invasion of axonal branches (Neumann et al., 2023c). The effects are parameter-dependent and shaped by the total electrical energy delivered (TEED), which governs the extent of neural tissue engagement. More recently, human intraoperative multiunit recordings in PD patients have revealed that excitatory cortico-STN inputs depress rapidly, while inhibitory GPe-STN inputs are relatively resilient, in contrast to striatal inhibitory inputs to SNr, which depress strongly. These findings underscore that DBS effects are not only anatomy-dependent but also synapse-specific, offering a mechanistic framework that links stimulation parameters and local microcircuit properties to observed clinical outcomes.

Still local but involving a large set of synaptic activity at the neuronal population level, intracranial field potential recordings have been proposed as a therapeutic target of DBS, reflected in robust oscillatory signatures of disease and DBS responses. In Parkinson’s disease, exaggerated beta activity (13–35 Hz) reflects the hypodopaminergic state and scales with motor severity (Lofredi et al., 2023), while effective DBS suppresses these oscillations in proportion to clinical benefit (Oswal et al., 2016). More recently, high-frequency stimulation has been shown to evoke resonant neural activity as so-called ERNA (Kara A Johnson et al., 2023) and gamma entrainment (Olaru et al., 2025), reproducible markers of dopaminergic tone. Beta suppression, gamma entrainment and ERNA illustrate that DBS not only alters firing of individual neurons but also reshapes mesoscale patterns of population activity, offering potential biomarkers for closed-loop control as an emergent technological advance.

#### 2.1.2 Distributed network effects of DBS

Importantly, the abovementioned local effects should not be misinterpreted to indicate that DBS primarily acts at the local level. In addition to local synaptic and mesoscale population effects, it exerts robust influences at the level of distributed brain networks. Suppression of pathological beta activity, for instance, is not confined to the STN or GPi but can also be observed across interconnected cortical and subcortical regions, as demonstrated using EEG, MEG and ECoG recordings (Binns et al., 2025, Oswal et al., 2016). Beyond beta suppression, DBS reduces exaggerated cortical beta–gamma cross-frequency coupling, a phenomenon linked to the parkinsonian state and thought to reflect abnormal nesting of broadband cortical activity into beta cycles (de Hemptinne et al., 2015). Detailed cycle-by-cycle analyses further suggest that DBS smooths the pathological waveform sharpness of beta oscillations, thereby preventing excessive coupling of broadband signals to beta troughs and peaks (Cole et al., 2017) . DBS can also induce stimulation-locked evoked responses in cortex at short latencies, reflecting rapid engagement of basal-ganglia–thalamo-cortical projections (Spooner et al., 2024) . More complex entrainment phenomena have been observed as well: finely tuned gamma oscillations in motor and premotor cortices can couple to the stimulation frequency and its harmonics during STN-DBS (Olaru et al., 2025), an effect reproduced by computational mean-field models and hypothesized to emerge from reciprocal excitatory–inhibitory circuits (Sermon et al., 2024). Together, these findings highlight that DBS not only suppresses pathological rhythms but also reshapes oscillatory coupling and resonance in large-scale cortical–subcortical networks. Recent multimodal work suggests that high-frequency beta rhythms originating in cortex are transformed into slower pathological beta within the STN–GPe loop, an aberrant resonance that DBS disrupts (Cagnan et al., 2019, Oswal et al., 2021). Finally, fMRI-based functional connectomics has shown that DBS can lead to a restoration of brain network activity patterns towards those seen in healthy controls, underlining the potential of DBS as a therapy capable of normalizing whole-brain network states (Horn et al., 2019b).

### 2.2 Emergent technological innovations for patient centered translation in deep brain stimulation

**Fig. 1:**
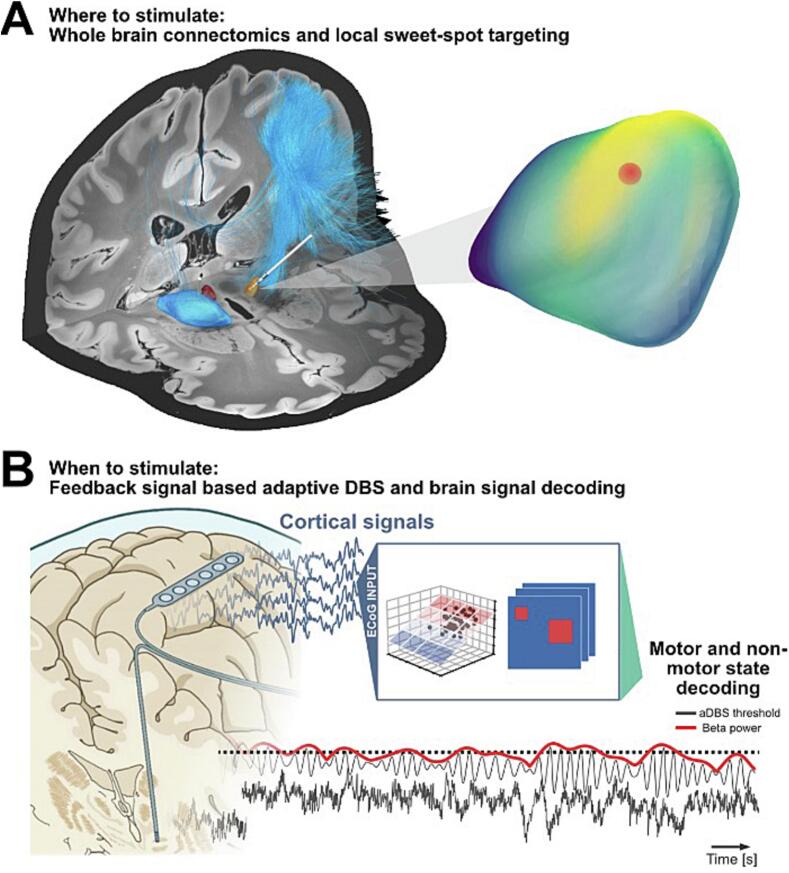
**Emergent technologies in invasive deep brain stimulation.** (a) Connectomic targeting: Structural and functional connectomics are used to guide DBS electrode placement beyond single anatomical nuclei, integrating whole-brain network architecture with local “sweet-spot” optimization. Tractography and network models identify stimulation sites whose engagement of distributed circuits best predicts clinical benefit. (b) Adaptive, biomarker-driven DBS: Cortical and subcortical brain signals are recorded and decoded in real time to infer motor and non-motor brain states. These neural biomarkers (e.g., spectral power or decoded states) are used as feedback signals to dynamically adjust stimulation parameters, enabling closed-loop DBS that adapts stimulation timing and intensity to ongoing brain activity.

The growing mechanistic understanding of DBS, from its local synaptic and mesoscale population effects to its influence on distributed cortical and subcortical networks, has laid the scientific foundation for the next generation of neuromodulation technologies. These insights have given rise to two major technological innovations: connectomic DBS, emerging from neuroimaging-centered research that maps the structural and functional architecture of therapeutic networks (Fig. 1A), and adaptive DBS, rooted in neurophysiology-centered research that harnesses real-time neural feedback signals for state-dependent stimulation control (Fig. 1B). Together, these approaches exemplify how fundamental research across disciplines has transformed DBS into a circuit-based, precision neuromodulation therapy.

#### 2.2.1 Towards automatized DBS programming from electrode localization to connectomic DBS

Connectomic DBS emphasizes the spatial dimension: by linking electrode locations with structural and functional connectivity, it has become possible to identify symptom-specific networks across patients. After postoperative imaging is co-registered to preoperative MRI, electrodes can be localized in stereotactic space and normalized to a standard template such as MNI using automated pipelines like Lead-DBS (Horn et al., 2019a, Neudorfer et al., 2023). This enables systematic comparison of stimulation sites across patients and facilitates group-level analyses within a shared anatomical framework (Treu et al., 2020). Within this framework, sweet-spot mapping can be used to identify local regions where stimulation is most consistently associated with clinical improvement. By correlating estimated volumes of tissue activated (VTAs) with quantitative outcome measures, probabilistic maps of beneficial (“sweet”) and detrimental (“sour”) regions can be derived (Dembek et al., 2019). To extend beyond local effects, fiber filtering can be applied using normative diffusion MRI connectomes derived from large healthy cohorts such as the Human Connectome Project (Li et al., 2020). Each VTA serves as a seed for whole-brain tractography, and fibers whose connectivity strength correlates with therapeutic response are isolated, delineating the structural pathways mediating clinical benefit or side effects. Similarly, functional connectivity mapping based on normative resting-state fMRI data enables each stimulation site to be linked to distributed brain networks (Horn et al., 2017). Voxel-wise correlations between connectivity and clinical outcomes reveal cortical and subcortical nodes whose coupling strength predicts treatment response. Analyses have first been performed on total improvement scores and later refined to symptom-specific subscores, showing distinct yet overlapping circuits for tremor, rigidity, or bradykinesia (Akram et al., 2017, Rajamani et al., 2024). While normative connectomics offers a robust framework for group-level mapping, several authors have developed precise methodologies at the individual level through subject-specific anatomy (Neudorfer et al., 2022), tractography (Coenen et al., 2019) or active DBS fMRI (Boutet et al., 2021). Together, these individualized mapping approaches complement normative models by providing a basis for patient-specific validation of DBS parameters and target engagement.

#### 2.2.2 Clinical translation of neurophysiological advances from biomarker mapping to adaptive DBS

Adaptive DBS extends precision neuromodulation into the temporal domain by adjusting stimulation in real time rather than delivering continuous high-frequency pulses. After decades of experimental research with externalized electrodes, the introduction of sensing-enabled implants has brought these approaches into clinical practice, allowing direct recording of neural activity from chronically implanted leads and enabling data-driven programming. But even during electrode implantation, biomarker mapping can already be used to identify electrophysiological signatures of target nuclei. Microelectrode recordings and local field potentials (LFPs) can characterize firing patterns, oscillatory dynamics, and spike–field relationships that are specific to functional subterritories within deep brain targets (Scherer et al., 2022, Zaidel et al., 2010). These measures can be used to functionally verify target engagement and to inform electrode placement beyond anatomical criteria. Following implantation, resonant activity characterization can be utilized to assess how test stimulation modulates local and distributed networks (Sinclair et al., 2021, Steiner et al., 2024). By recording spectral and temporal responses through LFPs or electrocorticography (ECoG), frequency-specific resonances can be identified that reflect network-level interactions and circuit mechanisms engaged by stimulation (Sermon et al., 2024, Steiner et al., 2024). For clinical programming, biomarker localization can be used to guide contact selection and parameter tuning (Neumann et al., 2017). Among current advances in neuromodulation, adaptive DBS (aDBS) represents one of the most exciting developments. Having evolved from an experimental research concept to a clinically available therapy, aDBS enables real-time, state-dependent modulation of brain activity by continuously monitoring neural signals and dynamically adjusting stimulation according to predefined feedback signal thresholds (Neumann et al., 2023a). In Parkinson’s disease, its feasibility and efficacy has been demonstrated in multicenter clinical trials, showing improved symptom control and reduced stimulation time compared to conventional continuous DBS (Bronte-Stewart et al., 2025, Oehrn et al., 2024). Recent developments extend these methods toward multivariate decoding of brain states (Cavallo et al., 2025, Merk et al., 2022a, Merk et al., 2025b, Neumann et al., 2019) . By integrating simultaneous cortical and subcortical recordings, machine learning models can be trained to decode behavioral or internal states in real time (Cavallo et al., 2025, Dixon et al., 2026, Merk et al., 2025b) . These models can then be used to dynamically adjust stimulation according to ongoing neural context, representing the next step toward intelligent, closed-loop neuromodulation.

### 2.3 Emergent clinical translation of neuroimaging and neurophysiological advancements in deep brain stimulation from movement disorders to neuropsychiatry

#### 2.3.1 Parkinson’s disease

Parkinson’s disease (PD) is the first and currently only FDA-approved indication for adaptive DBS (aDBS). The control signal underlying current systems is beta power (13–35 Hz) recorded from the subthalamic nucleus (STN), which accompanies slowness and rigidity, whereas dopaminergic medication or effective DBS suppresses this activity in parallel with motor improvement (Lofredi et al., 2023). This direct correspondence allows beta power to be used as a real-time biomarker of disease state, enabling stimulation amplitude to adjust automatically to moment-to-moment fluctuations in patient state (Neumann et al., 2023a). The multicenter ADAPT-PD trial provided pivotal evidence for long-term, at-home aDBS in PD (Bronte-Stewart et al., 2025). Participants alternating between continuous and adaptive modes showed longer periods in “on” motor states without troublesome dyskinesias and required less total stimulation time when stimulation was modulated by beta activity. Beyond beta modulation, entrained gamma activity (typically 60–90 Hz, often appearing at half the stimulation frequency) has emerged as a promising complementary feedback signal (Oehrn et al., 2024). This stimulation-entrained narrowband gamma increases with dopaminergic tone and correlates with clinical improvement during effective STN-DBS. Because it is phase-locked to the stimulation pattern and less susceptible to movement artifacts, entrained gamma may provide a more stable physiological readout of therapeutic engagement and support more reliable adaptive control across patients. Not all patients exhibit sufficiently clean or stable local field potentials for reliable feedback control. Movement artifacts, stimulation interference, or weak beta activity can limit performance (Neumann et al., 2021, Stanslaski et al., 2024). Cortical sensing electrodes over motor areas can improve signal quality and, when combined with subthalamic recordings, enable brain-signal decoding (Herron et al., 2025, Merk et al., 2022b). Machine-learning models can integrate these multimodal signals to estimate symptom severity in real time (Köhler et al., 2024, Lauro et al., 2023, Merk et al., 2025c) and potentially capture broader aspects of patient state, including behavior, motor fluctuations, and non-motor symptoms such as sleep or mood changes. Although still experimental, such approaches could transform chronic neural recordings into an objective, continuous measure of both motor and behavioral states in Parkinson’s disease. In parallel, connectomic DBS has advanced the spatial dimension of therapy in PD. Clinicians can identify which electrode contacts lie within functionally relevant dorsolateral motor domain and adjacent tracts such as pallidothalamic, hyperdirect, or cerebellothalamic fibers and adjust parameters to maximize engagement of therapeutic pathways while avoiding spread into associative or limbic territories. These visualization tools are now incorporated into clinical software environments and are increasingly used to guide postoperative programming and outcome prediction. Building on this framework, the Cleartune algorithm developed by Rajamani et al. introduces a data-driven approach that operationalizes network targeting for individual patients (Rajamani et al., 2024). By leveraging tractography-derived symptom–response libraries from large multicenter datasets, Cleartune identifies the white-matter tracts most predictive of improvement across distinct symptom domains such as tremor, rigidity, bradykinesia, and axial control and recommends stimulation settings that align with a patient’s dominant symptom profile. This approach moves beyond purely empirical programming toward quantitative, symptom-specific parameter optimization and is prospectively validated in a small cohort of patients.

#### 2.3.2 Essential tremor

Essential tremor (ET) is the most common movement disorder treated with DBS, typically targeting the ventral intermediate nucleus (VIM) or the dentatorubrothalamic tract (DRT). Recent technological advances have refined both imaging-based targeting and physiological characterization of tremor dynamics. High-resolution fast gray matter acquisition T1 inversion recovery (FGATIR) MRI that suppresses white matter signal and thus increases contrast in grey matter nuclei now enables direct visualization of a VIM DBS hotspot, promising to improve electrode placement accuracy and reductions in programming time (Neudorfer et al., 2022). In parallel, connectivity studies have demonstrated that stimulation sites with strong connectivity to primary motor cortex and cerebellum are associated with optimal tremor suppression (Goede et al., 2025). Beyond imaging, closed-loop and decoding approaches are emerging as next-generation strategies for tremor control. Multiple groups have now shown that tremor amplitude and movement state can be decoded in real time from local field potentials in the thalamus and motor cortex, paving the way for adaptive DBS systems that adjust stimulation only during active tremor episodes (Merk et al., 2022a). Such movement decoders may reduce stimulation time, minimize side effects, and personalize therapy delivery for people living with essential tremor.

#### 2.3.3 Dystonia

Dystonia is a hyperkinetic movement disorder characterized by involuntary, sustained, or intermittent muscle contractions that lead to abnormal postures and repetitive movements that can be treated with DBS targeting the internal globus pallidus (GPi) or subthalamic nucleus (STN). Multicenter analyses have revealed two distinct therapeutic networks underlying clinical improvement: one linking the basal ganglia to the primary motor cortex, associated with limb dystonia and blepharospasm, and another involving thalamic and cingulo-opercular regions, associated with cervical and axial forms (Butenko et al., 2025). Structural connectivity mapping further showed that modulation of hyperdirect and subthalamopallidal fibers benefits limb symptoms, whereas engagement of cerebellothalamic and pallidothalamic projections relates to axial improvement, supporting a network-based framework for symptom-specific targeting. At the physiological level, invasive recordings indicate that dystonia is characterized by enhanced low-frequency activity (3–12 Hz) within the basal ganglia, particularly along the striatopallidal pathway (Lofredi et al., 2024). This activity correlates with symptom severity and decreases with effective stimulation, identifying low-frequency synchronization as a potential biomarker for adaptive DBS. Recent chronic multisite recordings in patients with cervical dystonia have further shown that both low-frequency activity and narrowband gamma oscillations (∼60 Hz) in motor cortex track symptom fluctuations and are modulated by stimulation (Cernera et al., 2025). Reductions in low-frequency power and concurrent increases in stimulation-entrained gamma power were associated with clinical improvement, suggesting that gamma entrainment may serve as an additional marker of effective DBS. Integrating such physiological feedback with connectomic targeting could enable circuit-specific, closed-loop stimulation tailored to individual dystonia subtypes.

#### 2.3.4 Obsessive–compulsive disorder

Treatment-resistant obsessive–compulsive disorder (OCD) was among the first psychiatric DBS indications approved under humanitarian exemption. Current therapeutic targets, including the anterior limb of the internal capsule (ALIC), ventral striatum, and nucleus accumbens, converge on fronto–striatal–thalamic circuits implicated in cognitive control and reward processing. Beneficial connectivity consistently involves fibers projecting from the ALIC to the dorsomedial prefrontal and anterior cingulate cortices (Horn et al., 2026). At the physiological level, chronic intracranial recordings have revealed that activity in the theta–alpha range (4–12 Hz) within the ventral striatum and medial prefrontal cortex increases during anxiety and compulsive states and decreases during symptom relief or exposure therapy, suggesting its potential as a biomarker for adaptive DBS (Vissani et al., 2023). Continuous recordings during everyday life have revealed that this low-frequency activity follows circadian patterns and that the predictability of neural dynamics differentiates responders from non-responders to DBS (Merk et al., 2025a, Provenza et al., 2024). In patients with persistent symptoms, neural activity remains highly predictable, whereas in responders, predictability decreases as clinical symptoms improve. Preliminary closed-loop paradigms have demonstrated that stimulation triggered by these state-dependent features can reduce symptom severity while minimizing stimulation time, indicating the feasibility of adaptive, biomarker-driven DBS for OCD.

#### 2.3.5 Tourette syndrome

DBS in Tourette syndrome has been applied to thalamic, pallidal, and internal capsule targets, with clinical benefits linked to suppression of tics and improvement in associated behavioural symptoms. Connectomic mapping shows that effective stimulation sites converge on cortico-striato-thalamo-cortical loops, particularly those connecting motor and prefrontal cortices with striatal regions (Kara A. Johnson et al., 2023). These findings reinforce the principle that symptom relief depends on modulation of distributed circuits that mediate tic generation and premonitory urges. Neurophysiological work has identified tic-related oscillatory activity in thalamic and pallidal structures (Neumann et al., 2018), which may serve as candidate biomarkers for adaptive paradigms that trigger stimulation in response to imminent tic expression (Cernera et al., 2022).

#### 2.3.6 Depression

Deep brain stimulation (DBS) of the subcallosal cingulate (SCC) has emerged as a promising therapy for treatment-resistant depression (TRD), capable of providing sustained symptom relief in patients unresponsive to medication or psychotherapy. While the first large multicenter trial has not reached its primary endpoint, recent work using chronically implanted sensing-enabled DBS systems has begun to uncover objective physiological markers of recovery (Alagapan et al., 2023). In a 24-week clinical study, SCC-DBS produced robust improvements, with 90% of patients responding and 70% achieving remission. Analysis of chronic SCC local field potentials identified a stable, patient-specific electrophysiological signature of mood state that distinguished sustained clinical recovery from transient fluctuations. Variability in treatment trajectories was linked to differences in the structural integrity and functional connectivity of the targeted SCC white-matter network. Another study found that perceived emotions in depressed patients can be accurately decoded from limbic network hubs in the SCC within 600 ms after stimulus onset, with decoder performance being correlated with DBS response (Merk et al., 2025b). These findings demonstrate that objective, brain-based feedback signals can guide adaptive DBS for depression, enabling personalized adjustments based on neural state rather than subjective symptom reporting.

#### 2.3.7 Alzheimer’s disease

DBS of the fornix is an investigational therapy for mild Alzheimer’s disease (AD) that aims to restore activity within memory-related limbic circuits. Clinical outcomes have been variable, with cognitive improvement in some patients and decline in others. Connectomic analyses of multicenter trial data indicate that stimulation of the circuit of Papez and stria terminalis, key pathways linking hippocampus, thalamus, and cingulate cortex, is associated with cognitive benefit, defining an optimal network target at their interface (Ríos et al., 2022). Functional connectivity to hippocampal–cingulate networks further predicts favorable outcomes, suggesting that precise electrode placement within these memory circuits is crucial for efficacy. Comparative studies across Parkinson’s and Alzheimer’s disease show that the cognitive effects of DBS depend on hippocampal connectivity and patient age (Howard et al., 2025) . Hippocampal engagement improves cognition in AD but worsens it in PD, likely reflecting disease-specific network integrity. Older AD patients with preserved limbic structure appear to benefit most, highlighting the importance of personalized, connectomics-guided targeting in future DBS applications for dementia.

### 2.4 *Conclusion and current knowledge gaps*

The fusion of connectomic and adaptive deep brain stimulation (DBS) defines the next stage of precision neuromodulation. Connectomic DBS identifies which circuits to stimulate, while adaptive DBS determines when to stimulate based on real-time neural dynamics. Integrating these approaches could enable symptom decoding and circuit-specific modulation in real time, transforming DBS into an intelligent, closed-loop therapy. This framework, recently termed adaptive circuit targeting (Horn and Neumann, 2025), could aim to decode neural states continuously and direct stimulation toward the most relevant network nodes for each patient. However, several key knowledge gaps remain before such a vision can be fully realized. The mechanistic underpinnings linking stimulation parameters to large-scale network reconfiguration are still incompletely understood, and the causal hierarchy between local and distributed effects remains to be resolved. Long-term neuroadaptive changes induced by chronic stimulation, including how the brain reorganizes under sustained closed-loop control, are also largely unknown. Furthermore, the generalizability of biomarker-based adaptive strategies across patients, disease states, and recording modalities has yet to be systematically validated. Technical and regulatory challenges persist in ensuring interoperability between devices, standardization of data formats, and the integration of multimodal datasets for individualized modeling. Finally, ethical and clinical questions regarding algorithmic autonomy, data privacy, and patient agency in AI-driven neuromodulation demand careful interdisciplinary dialogue. By addressing these open questions, the field can move toward a unified framework in which circuit-based, biomarker-guided interventions are delivered through the least invasive yet most effective means available. Transcranial ultrasound neuromodulation (TUS) for example, is a deep brain stimulation technique that could be developed in a way to leverage the same principles of adaptive and connectomic DBS, without requiring brain surgery. The convergence of invasive and non-invasive modalities thus defines the next frontier in therapeutic clinical neurophysiology. In the following, we will describe the emergent technological advances in non-invasive neuromodulation with a special emphasis on methods utilizing ultrasound.

## 3 Clinical Applications of Low Intensity Focused Transcranial Ultrasound Stimulation as an Emergent Non-Invasive Deep Brain Stimulation Technique

### 3.1 Principles and Mechanisms

Building on mechanistic and translational insights from deep brain stimulation, there is growing interest in non-invasive methods that can engage the same circuits without the risks of surgery or implanted hardware. Among these, transcranial ultrasound stands out as uniquely capable: unlike electrical or magnetic approaches, it can reach both superficial and deep targets with millimeter precision (Darmani et al., 2022). Deep penetration makes ultrasound the closest non-invasive analogue to DBS. Like DBS, TUS modulates neuronal populations locally and at the network level. Several candidate mechanisms explain how ultrasound modulates neuronal and circuit activity (Kamimura et al., 2020, Tyler et al., 2018). Mechanical effects are considered central at the low intensities used in transcranial ultrasound stimulation (TUS). Cyclical acoustic pressure (compression/rarefaction at the carrier frequency) generates rapid particle displacements that periodically stretch and compress cell membranes (an “AC” like load), while acoustic radiation force, as a net “DC” load produces slower, sustained deformations over the duration of a pulse train. Together, these oscillatory (AC) and bias (DC) components alter membrane tension and can modulate ion channels. Experimental evidence supports direct gating of mechanosensitive potassium channels (K2P/TREK-TRAAK) that closely matches canonical mechanical stimulation (Sorum et al., 2021), modulation of Piezo channels, and facilitation of voltage-gated sodium and calcium currents (Tyler et al., 2018). These effects are reversible and parameter-dependent, consistent with observations that TUS can either enhance or suppress neural excitability depending on stimulation protocol design. A complementary hypothesis is the bilayer sonophore (intramembrane cavitation) model (Kamimura et al., 2020). In this view, the lipid bilayer, about 5 nm thick and electrically insulating, separates the conductive intracellular and extracellular fluids and therefore behaves like a small capacitor. Nanometer scale oscillations in response to the acoustic field slightly change membrane thickness and area, which in turn changes its electrical storage properties (capacitance) over time. As capacitance fluctuates, small displacement currents arise that can nudge the membrane voltage toward or away from spike threshold even when ion channels remain closed. Consequently, subthreshold ultrasound, meaning exposures below the level that directly open channels or trigger action potentials, may still shift excitability by gently biasing the membrane potential (Krasovitski et al., 2011, Plaksin, 2014, Plaksin et al., 2016). In addition, glial contributions have been proposed: astrocytic TRPA1 activation can trigger glutamate release via Best1 channels, suggesting that ultrasound may influence neural circuits indirectly through neuron–glia signaling (Kamimura et al., 2020). Although typically secondary, thermal effects of TUS also remain relevant. Even moderate temperature increases, on the order of 0.5–1 °C in brain tissue, can alter ion channel kinetics and synaptic transmission. Such changes are well documented in peripheral nerves, animal models and may also occur in the human brain under certain sonication conditions (Darmani et al., 2022, Murphy and Fouragnan, 2024). To minimize heating, most neuromodulation protocols use pulsed low-intensity ultrasound with short pulse durations, especially given the skull’s tendency to absorb and re-radiate energy. Nonetheless, subtle thermal shifts likely contribute alongside mechanical pathways.

TUS outcomes also depend on physiological state and anatomical features. Baseline brain state, anesthesia, and ongoing activity can all influence whether ultrasound excites or inhibits neurons. In humans, applying the same theta burst TUS (tbTUS) protocol (Zeng et al., 2022) to two deep cortical targets produced different effects: posterior cingulate showed a localized decrease in GABA, whereas dorsal anterior cingulate did not, highlighting target-specific outcomes under the same exposure pattern (Yaakub et al., 2023). Complementing this, macaque work using matched parameters shows bidirectional, state-dependent effects: suppression of tactile-evoked activity during active sensory drive versus activation in connected regions at rest, and distinct dose–response curves in resting versus activated states (Yang et al., 2022). Skull thickness, morphology and tissue stiffness further affect the effective dose delivered (Attali et al., 2025a). Moreover, auditory and somatosensory co-stimulation (Kop et al., 2023) can confound readouts and should be controlled with masking and off-target active shams.

In sum, the emerging consensus is that TUS exerts its neuromodulatory effects through an interplay of mechanical strain, modest thermal shifts, and supporting glial and vascular responses. Understanding this interplay provides the mechanistic foundation for developing safe and effective protocols, while also guiding the technological and clinical advances that follow.

### 3.2 Emergent technological innovations in TUS transducer hardware and design

Just as DBS hardware evolved from simple constant-output stimulators to adaptive, sensing-enabled platforms, TUS is undergoing a parallel progression, from single-element sources toward neuronavigated arrays with aberration correction, electronic steering, and sophisticated helmet arrays. Many of the principles behind connectomic DBS, identifying symptom-relevant circuits, optimizing structural and functional engagement, and integrating individualized imaging, are now informing TUS targeting. New array designs allow ultrasound to be steered toward the same network-defined nodes previously accessible only with DBS. At its core, an ultrasound transducer converts electrical energy into mechanical pressure waves through piezoelectric crystals. When an alternating voltage is applied, the crystal oscillates, generating ultrasonic vibrations that couple into biological tissue. By adjusting frequency, aperture size, and driving patterns, transducers can tune the depth, focus, and intensity of energy delivered into the brain. A central challenge in TUS is the skull’s tendency to distort and attenuate the beam. The irregular thickness and heterogeneous composition of cranial bone cause phase aberrations that can shift the focal point, reduce intensity at the target, and increase off-target exposure (Attali et al., 2025a). Most published studies, especially those using single-element devices or basic arrays, do not implement real-time phase aberration correction. Instead, they rely on external simulations and modeling to approximate focus. Only a small number of recent systems incorporate built-in correction. In the following, we review these technologies:

#### 3.2.1 Single-element transducers

**Fig. 2:**
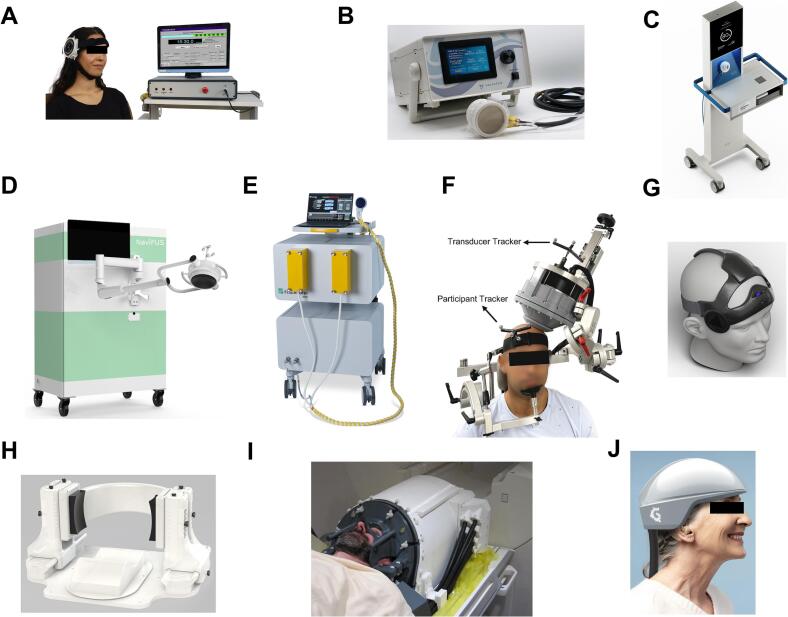
**Emergent innovations in TUS hardware.** Representative examples of TUS systems spanning single-element, annular, and phased-array transducers to next-generation helmet designs. These devices illustrate the field’s evolution from basic, fixed-focus setups toward neuronavigated and adaptive arrays with built-in or hardware-embedded aberration correction, enabling increasingly precise and personalized neuromodulation across clinical and research settings. (A) BrainSonix single-element MR-compatible transducer, (B) BrainBox NeuroFUS 4-element annular array, (C) SONOMIND device using *meta*-lens correction, (D) NaviFUS neuronavigated 256-element array, (E) Fraunhofer 256-element phased array, (F) Sonic Concepts model H317 128-element system, (G) Attune dual temporal wearable array, (H) Diadem 256-element system with real-time aberration correction, (I) NeuroHarmonic semi-ellipsoidal 256-element MRI-compatible helmet, and (J) Grey Matter custom-fit wearable helmet for repeated outpatient use.

Early neuromodulation studies relied on single focused transducers. These devices are technically simple and relatively inexpensive, producing a fixed focal spot defined by their geometry and frequency. They remain useful for proof-of-concept experiments, particularly where the target is superficial or precise steering is not required, unless paired with MRI for online targeting or using an initial MRI or CT scan with offline methods such as optical tracking and pre-TUS simulations using k-Wave (Treeby and Cox, 2010), BabelBrain (Pichardo, 2023) or similar planning software and techniques (Aubry et al., 2022). A representative device in this category is BrainSonix (BX Pulsar 1002, https://brainsonix.com/; Badran et al., 2020, Barksdale et al., 2025, Chou et al., 2025), which is fully MR-compatible and has the most published concurrent TUS-fMRI studies using this device (Fig. 2A).

#### 3.2.2 Annular arrays

Annular arrays consist of concentric rings that can be driven with relative delays, enabling variable focal depth and limited axial steering without the complexity or cost of large phased arrays. Compared with single-element transducers, they provide greater control over focal depth while retaining relatively simple hardware. They are useful in early translational studies where some flexibility is needed but full 3D steering is not required. Similar to single-element transducers, these devices also require pairing with MRI for online targeting, or with an initial MRI or CT scan combined with offline modeling methods for phase aberration correction, in conjunction with optical tracking during TUS. Some representative examples include the early-generation Sonic Concepts/BrainBox 4-element (NeuroFUS) transducers (https://brainbox-neuro.com/; Darmani et al., 2025, Yaakub et al., 2023) (Fig. 2B).

#### 3.2.3 Meta-lenses

A very recent study demonstrated a hardware-embedded correction strategy based on numerical modelling and calibration, introducing patient-specific 3D-printed “metalenses” that correct skull aberrations for single-element transducers, without requiring online MRI (Attali et al., 2025b). This technology is currently implemented in the SONOMIND device (https://www.sonomind.com/) (Fig. 2C). Meta-lenses are usually paired with single-element transducers (Maimbourg et al., 2018), though in principle they could complement annular arrays for added lateral control to further shape or steer the beam.

#### 3.2.4 Moderate aperture and flat phased arrays

The advent of multi-element phased arrays marked a turning point in ultrasound neuromodulation. By driving each element with independent phase and amplitude, these systems can electronically steer and shape the beam, correcting for some skull-induced distortions and reaching deep structures with greater precision. Similarly, they can be operated outside the MRI environment, using an initial MRI or CT scan combined with optical neuronavigation to target and compensate for phase aberrations via simulations. Representative devices include NaviFUS (https://navifus.com/; Lee et al., 2022) (Fig. 2D) and Fraunhofer phased arrays (Tretbar et al., 2025) (Fig. 2E), which both employ 256 elements, and the Sonic Concepts model H317, a 128-element phased array (https://sonicconcepts.com/; Zadeh et al., 2024) (Fig. 2F). There is also a wearable dual-array platform for home use, designed by Attune Neurosciences (https://www.attuneneuro.com/; Fan et al., 2024). This transducer employs 128 elements via two transducers interfacing with the temporal window to precisely target deep structures (Fig. 2G). The device implements MRI guided phase aberration correction and has ongoing developments towards device self-registration using ultrasound imaging. To date, only one device, Diadem (a 256-element array), has implemented real-time aberration correction without requiring MRI during operation with available published clinical data (https://spire.us/; Riis et al., 2024a, Riis et al., 2024b, Riis et al., 2025). Diadem uses paired transducers to generate interference patterns that dynamically adjust to skull properties (Fig. 2H). It should be noted that the NaviFUS Model 101 is also undergoing key development to incorporate real-time positional data from neuronavigation systems for dynamic aberration correction. This technology is currently being evaluated in clinical trials, although no published data are available at this time.

#### 3.2.5 Large aperture phased arrays – Helmets

The latest generation of TUS devices are helmet-shaped arrays designed to cover large cranial surfaces with hundreds of elements. By maximizing the aperture size and distributing emitters around the skull, these systems achieve exceptional focal precision. A landmark example is the 256-element semi-ellipsoidal helmet developed by Martin and colleagues, capable of producing a −3 dB focal spot as small as ∼1.3 mm lateral × 3.4 mm axial in free water and ∼1.5 × 1.5 × 2.2 mm in skull-corrected “in-situ” conditions (Martin et al., 2025) (Fig. 2I). This system integrates real-time fMRI compatibility, stereotactic positioning, and individualized treatment planning, enabling precise modulation of deep nuclei; this technology is currently implemented in the NeuroHarmonic device (https://neuroharmonics.com/). Similar in form to the long-established Exablate Neuro helmet for MR-guided high-intensity focused ultrasound (HIFU) ablation, which requires a minimally invasive stereotactic frame fixed with skull pins, the current device replaces this with a custom 3D-printed face and neck mask that stabilizes the head non-invasively and avoids the need for shaving. Like clinical ablation helmets, it uses CT- or MR-based acoustic models to pre-compute phase delays and applies electronic focusing, but it must still be operated inside an MRI scanner for navigation and online correction (Martin et al., 2025). Very recently, Grey Matter Neurosciences introduced a new helmet transducer that does not require MRI operation, using a custom-fit design derived from each patient’s brain scans (https://greymatterneurosciences.com/) (Fig. 2J). This ensures the helmet locks into the same position on the skull for each session, enabling precise targeting without external frames or online imaging and does not require subject immobilization. Once fabricated, the helmet can be worn repeatedly to reliably reach the same targets, even outside hospitals. It should be noted that element count and focal size for this device are not yet disclosed, limiting direct comparison to the 256-element semi-ellipsoidal helmet developed by Martin and colleagues.

In summary, precision and correction capacity scale with the number of elements and level of personalization, but so do complexity and cost. MRI integration provides the highest accuracy, yet it remains a major bottleneck, limiting advanced systems to specialized, resource-intensive settings. New approaches that bypass online MRI such as Diadem (Riis et al., 2024a), meta-lens-based designs (Attali et al., 2025b), and helmet-based designs (Grey Matter Neurosciences design), offer a pathway toward broader accessibility and clinical practicality. Importantly, all of these systems operate within low-intensity regimes (typically ISPPA ≤ 100 W/cm^2^, MI ≤ 1.9), well below tissue damage thresholds used in ablative HIFU, reinforcing their safety for repeated applications in neuromodulation. Several of these systems are already undergoing early-phase clinical trials in pain (Diadem, Attune Neuroscience), epilepsy (NaviFUS), Movement Disorders (Fraunhofer, Sonic Concept H317) and depression (SONOMIND, BrainBox), highlighting their translational momentum. Looking ahead, hybrid solutions that merge electronic steering, acoustic lensing, and computational modeling may bridge the divide between laboratory-grade precision and everyday usability. Beyond focal size and element number, practical issues such as device setup time, patient immobilization, and compatibility with concurrent monitoring (EEG, fMRI, MEG) will ultimately determine clinical uptake. Eventually, the field’s progress will hinge not only on engineering refinements but also on demonstration of reproducible neuromodulatory effects in controlled trials, paving the way for standardized therapeutic protocols.

### 3.3 Emergent Clinical Translation of TUS from Movement Disorders to Neuropsychiatric Disorders

The field increasingly recognizes that the future of non-invasive neuromodulation will rely on the same principles that revolutionized DBS namely connectomic network targeting, biomarker-guided feedback and state-dependent stimulation. Recent TUS studies that incorporate real-time imaging, closed-loop acoustic feedback, DBS-informed stimulation strategies, and AI-based control reflect a similar trajectory toward adaptive, network-based precision therapy. Many of the same disorders treated with DBS e.g. Parkinson’s disease, essential tremor, OCD, depression, epilepsy etc. are now being explored with TUS as a non-invasive alternative. This parallel clinical development reflects the shared underlying principle: modulating well-defined distributed circuits whose dysfunction drives symptoms.

One of the main directions for therapeutic translation that is being most actively and systematically pursued is in movement disorders. Investigations have begun and continue to explore the therapeutic effects of TUS applied to distinct nodes of the motor network that are pathophysiologically implicated in these conditions. Similar to movement disorders TUS is increasingly being tested in psychiatric populations, motivated by its ability to reach deep limbic and striatal nodes that previously required invasive or indirect modulation (Attali et al., 2025a, Davidson et al., 2025). Early human studies, though heterogeneous in design, converge on three consistent themes (Davidson et al., 2025): First, target specificity matters: clinical and biomarker effects depend on whether limbic hubs such as the amygdala and subcallosal cingulate cortex (SCC), thalamic nuclei, or striatal circuits are engaged. Second, dose and repetition shape durability: single sessions often show acute target engagement, whereas multi-session protocols are needed to consolidate clinical change. Third, safety has been consistently favorable, with no major adverse events reported under low-duty cycle, sub-thermal conditions. The next phase will require larger sham-controlled randomized clinical trials to establish durability and define optimal dosing and targeting approaches. This section reviews early clinical evidence in movement disorders, psychiatric conditions, chronic pain, epilepsy, and Alzheimer's disease, highlighting TUS's capacity for frequency-specific, network-level modulation under sub-thermal conditions. These studies highlight how flexible TUS can be; it can reach key brain regions safely, while also opening the door to more personalized approaches guided by real-time biomarkers.

#### 3.3.1 Parkinson’s Disease

**Fig. 3:**
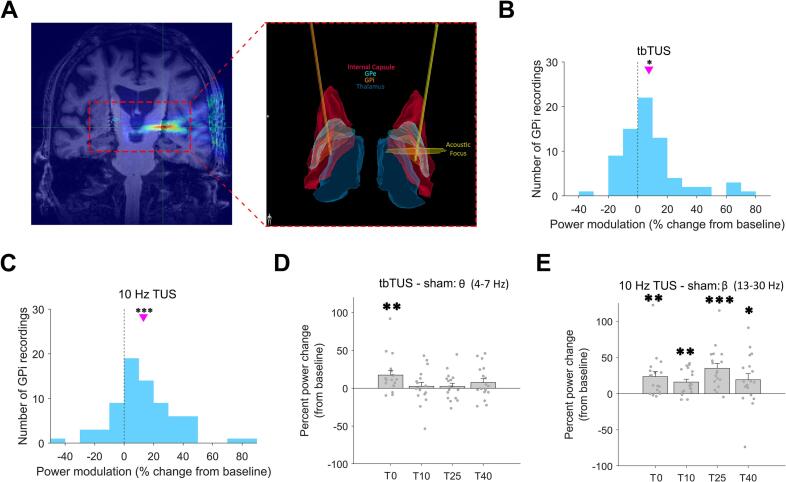
Parameter-specific modulation of pallidal oscillations by TUS in Parkinson’s disease. (A) Individualized acoustic simulations and 3D reconstructions showing targeting of the globus pallidus internus (GPi) via implanted DBS leads and modeled acoustic focus. (B–C) Distributions of GPi power changes from baseline following theta-burst (tbTUS) and 10 Hz TUS, showing increases in spectral power. (D–E) Group-level comparisons revealing that tbTUS selectively enhanced theta (4–7 Hz) activity, while 10 Hz TUS increased beta (13–30 Hz) power during and up to 40 min after stimulation compared to the sham conditions. Together, these results demonstrate frequency-dependent engagement of deep basal ganglia circuits by TUS in patients with Parkinson’s disease (adapted with permission from Darmani et al., 2025).

Early clinical studies suggest that TUS can engage canonical surgical and DBS targets in movement disorders and produce measurable and sometimes sustained physiological and behavioral effects, with responses that vary depending on target, stimulation protocol, and disease state. In a recent pivotal study in patients with Parkinson’s disease implanted with GPi-DBS electrodes, invasive brain signals were recorded as local field potentials (LFP) before, during, and after individualized TUS applications to the implanted leads (Fig. 3A). While both active protocols (theta-burst TUS [tbTUS] and 10 Hz TUS) increased power (Fig. 3B-C), no such changes were observed under either active or passive sham conditions. Most importantly, tbTUS and 10 Hz TUS protocols elicited distinct, frequency-dependent modulations of pallidal activity: enhancing theta oscillations during tbTUS and beta activity during and for up to 40 minutes after 10 Hz stimulation (Fig. 3D-E).

**Fig. 4:**
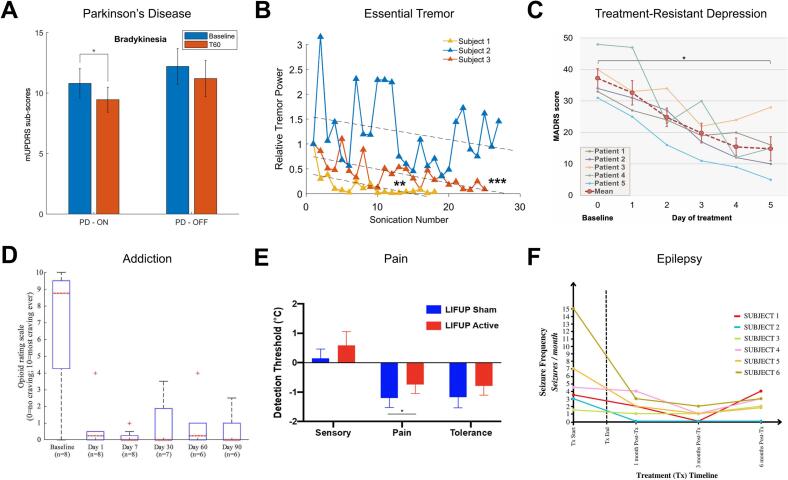
Exemplary clinical applications of TUS in brain disorders. (A) Parkinson’s disease: Theta-burst TUS of the motor cortex produced significant clinical improvement, reducing bradykinesia and enhancing motor performance in PD patients (Adapted with permission from Grippe et al., 2024) (B) Essential tremor: Low-intensity focused TUS of the thalamic VIM markedly reduced tremor amplitude (up to 97–98%) with rapid onset and cumulative improvement, demonstrating reversible tremor suppression (Adapted with permission from Riis et al., 2024d). (C) Treatment-resistant depression: Personalized metalens-corrected TUS targeting the subcallosal cingulate produced rapid antidepressant effects, with mean MADRS reduction of ∼ 61% by Day 5 (Adapted with permission from Attali et al., 2025b). (D) Addiction: MRI-guided TUS of the bilateral nucleus accumbens reduced cue-induced craving by 91% and maintained abstinence in most participants for 90 days, reflecting modulation of reward circuitry (Adapted with permission from Rezai et al., 2025). (E) Pain: MRI-guided TUS of the anterior thalamus attenuated pain sensitization, reducing the decline in thermal pain thresholds by ∼ 50% compared with sham (Adapted with permission from Badran et al., 2020). (F) Epilepsy: Repeated hippocampal TUS in drug-resistant temporal lobe epilepsy reduced seizure frequency by ∼ 50% (Adapted with permission from Bubrick et al., 2024).

These effects were absent under both sham conditions and scaled with dopaminergic medication dose, providing the first direct electrophysiological evidence that TUS can selectively engage and modulate deep basal ganglia circuits in a parameter-specific manner (Darmani et al., 2025). Extending this approach, simultaneous STN LFP recordings showed that tbTUS to the motor cortex (M1) versus GPi induced dissociable network effects (M1: ↑θ, ↓β; GPi: ↑β), persisting for ∼45 min and amplified during movement, evidence that TUS can bidirectionally tune pathological β rhythms along the cortico-basal ganglia loop (Sarica et al., 2025). At the cortical level, tbTUS to M1 increased corticospinal excitability in PD under an accelerated session design, though single-session clinical scores did not shift, suggesting a role as a dosing and feasibility signal for future multi-session trials (Samuel et al., 2023). Notably, medication state of PD patients appears critical in TUS efficacy: in a randomized case-controlled study, M1 tbTUS produced LTP-like facilitation in controls and in PD patients ON-medication, but not OFF-medication, and modestly improved bradykinesia at 60 min in the on-state, consistent with dopamine-dependent plasticity gating (Grippe et al., 2024) (Fig. 4A).

#### 3.3.2 Essential Tremor

In essential tremor (ET), small but convergent studies targeting the ventral intermediate nucleus (VIM) or the dentato-rubero-thalamic tract (DRT) circuits report rapid and sometimes pronounced reductions in tremor amplitude. MR-guided low-intensity thalamic TUS with aberration correction achieved substantial accelerometry-verified tremor power reductions (often> 90%) in responders, with negligible MR thermometry changes, supporting a predominantly mechanical mechanism (Bancel et al., 2024). VIM sonications also nearly abolished tremor in 2/3 patients within seconds, with gradual washout, demonstrating on-target, reversible modulation (Riis et al., 2024d) (Fig. 4B). Outside the scanner, clinically meaningful improvements on the Essential Tremor Rating Assessment Scale (TETRAS) were observed in all 10 participants of an open-label, repeated TUS to the VIM, with no adverse events reported, supporting pragmatic feasibility and motivating controlled trials (Deveney et al., 2024). Finally, phased-array, multi-focus strategies are being piloted to mitigate tracking and targeting error and to stabilize tremor benefits across VIM neighborhoods in ET and parkinsonian tremor (Kiss et al., 2025).

#### 3.3.3 Depression

Several groups have targeted canonical DBS and TMS regions in treatment-resistant depression (TRD) with TUS. In one sham-controlled crossover study, bilateral SCC sonication produced fMRI-based target-specific deactivation and yielded greater improvements in sadness ratings and HDRS6-6 score relative to sham (Riis et al., 2025). (Barksdale et al., 2025) extended this approach to the amygdala, demonstrating in a double-blind fMRI paradigm that active sonication reduced left amygdala activity relative to sham, followed by an open-label course of 15 daily sessions in which amygdala-targeted TUS produced robust symptom reductions and attenuated amygdala reactivity to emotional faces. A case-level study using cross-beam phased arrays to target the anterior thalamus (ANT) reported rapid within-day improvements on depression scales, along with suppression of default mode network (DMN) hyperconnectivity and these effects were not observed with ventral capsule (VC) stimulation (Fan et al., 2024). An individualized SCC targeting approach using metalens-corrected portable devices was recently evaluated in five patients with TRD. This intensive five-day, 25-session protocol produced a 60% mean reduction in Montgomery–Åsberg Depression Rating Scale (MADRS) scores, with four responders and no adverse events (Attali et al., 2025b) (Fig. 4C). Finally, a randomized controlled pilot trial applying a 2- week transcranial pulse stimulation (TPS) to the left dorsolateral prefrontal cortex (DLPFC) demonstrated significant improvements on the 17-item Hamilton Depression Rating Scale (HDRS-17) compared to a control group, with effects sustained for three months (Cheung et al., 2023). Collectively, these studies show that TUS can acutely modulate deep affective hubs and, when applied repeatedly, yield clinically meaningful antidepressant effects.

#### 3.3.4 Addiction

Rezai, Mahoney, and colleagues pioneered the clinical translation of TUS for substance use disorders, focusing on the nucleus accumbens (NAc) (Mahoney et al., 2023b, Mahoney et al., 2023a, Rezai et al., 2025). In open-label feasibility trials, MRI-guided low-intensity sonications of the nucleus accumbens using the Insightec low-frequency Exablate Neuro system reduced cue-induced craving and substance use in individuals with alcohol and opioid use disorders, with a favorable safety profile and no reported seizures or psychiatric adverse events (Mahoney et al., 2023b, 2023a). Physiological readouts included cue-induced craving reduction as the primary measure, with urine toxicology serving as an objective physiological correlate. Subsequent replication studies in individuals with severe opioid use disorder demonstrated ∼90% reductions in cue-induced craving, accompanied by urine toxicology–confirmed abstinence (7 of 8 at 30 days; 5 of 8 at 90 days) (Rezai et al., 2025) (Fig. 4D). Resting-state fMRI further revealed decreased connectivity between the NAc and reward-control regions (vmPFC, ACC, dlPFC), indicating circuit-level modulation. While sham-controlled trials are still pending, these early results highlight the potential of NAc sonication as a non-invasive analog to DBS for addiction, directly engaging reward valuation circuits that remain inaccessible to TMS.

#### 3.3.5 Pain

Chronic pain remains one of the most difficult conditions to treat, with mixed results from invasive DBS and SCS and limited pharmacological options. Early studies suggest that TUS can noninvasively produce measurable reductions in pain intensity and interference via both sensory (S1, thalamus) and affective (ACC) components of pain. A double-blind, sham-controlled study tested whether MRI-guided TUS targeting the right anterior thalamus could modulate thermal pain sensitivity in healthy adults. The authors found that active TUS significantly attenuated thermal pain sensitivity compared to sham, demonstrating an acute antinociceptive effect of anterior thalamic stimulation (Badran et al., 2020). This approach has been extended to patients with chronic pain syndromes, reporting reductions in pain scores after TUS targeted the anterior cingulate cortex (ACC) comparing two 40-minute active vs. sham sessions (Riis et al., 2024c) (Fig. 4E). Active stimulation produced large and lasting pain reductions (up to ∼60% immediately and ∼33% at day 7), far exceeding sham and with no adverse events. The ACC has also been targeted in patients with refractory neuropathic pain, with repeated bilateral sessions delivered over a two-week period (Shin et al., 2023). Pain ratings on the visual analog scale and interference scores on the Brief Pain Inventory improved significantly at four weeks, without adverse events or MRI changes.

#### 3.3.6 Epilepsy

Epilepsy is an area of active investigation for TUS, driven by the need for noninvasive approaches to control seizures in drug-resistant epilepsy (DRE). Early human studies have primarily targeted the hippocampus and anterior temporal lobe, the canonical seizure-onset zones (SOZ) in mesial temporal lobe epilepsy (mTLE). Initial safety testing involved delivering sonications to the anterior temporal lobe in patients undergoing planned resections (Stern et al., 2021). Sonications performed under MRI guidance showed no histological damage in resected tissue and no consistent neuropsychological decline, establishing the feasibility of sub-thermal exposure. Building on this, a pilot safety and feasibility trial applying serial hippocampal TUS in six adults with mTLE was conducted. Across six sessions over three weeks, five patients experienced seizure reductions averaging ∼50%, and one remained seizure-free for more than a year (Bubrick et al., 2024) (Fig. 4F). Resting-state functional MRI in a subset revealed improved default mode network organization, which correlated with clinical improvement. TUS has also been applied directly over the SOZ in patients with DRE undergoing stereo-electroencephalography (SEEG). Using a neuronavigated multi-element system, they recorded intracranial EEG during and after stimulation, demonstrating significant spectral-power changes at the epileptogenic focus without adverse events, supporting both safety and physiological engagement across multiple sessions (Lee et al., 2022).

#### 3.3.7 Alzheimer’s disease

Low-intensity ultrasound for Alzheimer’s disease was pioneered through the development of transcranial pulse stimulation (TPS), a distinct neuromodulation approach using the CE-marked NEUROLITH system (Storz Medical AG, https://www.storzmedical.com/en/; Beisteiner et al., 2020, Beisteiner and Lozano, 2020). TPS delivers ultrashort single pulses (∼3 µs), avoiding standing waves and excess heating seen with long sonication trains in TUS, and enables MRI-navigated stimulation of cortical and deep targets. Across clinical studies, TPS has shown consistent symptomatic benefits in AD (Chen et al., 2024). Early open-label investigations showed memory and language improvements lasting up to three months, paralleled by increased hippocampal connectivity on fMRI (Beisteiner et al., 2020). Follow-up analyses demonstrated reduced cortical atrophy in default mode regions and alleviation of depressive symptoms linked to normalization of salience–ventromedial network interactions (Cheung et al., 2023, Popescu et al., 2021). Most recently, a randomized sham-controlled trial in 60 patients confirmed safety and feasibility, with younger patients (<70 years) showing robust cognitive gains and network upregulation (Matt et al., 2025). Together, these findings suggest that TPS can induce functional and structural plasticity capable of stabilizing cognition and mood in Alzheimer’s disease, offering a promising add-on where established therapies remain limited (Cont et al., 2022, Matt et al., 2022).

### 3.4 Current knowledge gaps

Despite the rapid progress outlined above, several fundamental limitations continue to constrain the clinical translation of TUS. First, the parameter space remains vast and insufficiently standardized. Frequency, pulse duration, duty cycle, intensity, repetition rate, and sonication duration can be combined in numerous ways, each associated with distinct and sometimes nonlinear biophysical and neurophysiological effects. While different parameter regimes may preferentially engage mechanical versus thermal mechanisms or favor transient versus longer-lasting plasticity, systematic dose–response relationships remain poorly defined. This lack of standardization and incomplete reporting limits reproducibility, complicates cross-study comparisons, and precludes the rational design of reliable, bidirectional (e.g., excitatory vs. inhibitory) stimulation protocols.

Second, both target- and subject-specific factors introduce substantial variability. TUS effects are shaped by local cytoarchitecture, receptor expression, ion channel composition, and baseline network state, in addition to skull-related acoustic distortions. Inter-individual differences in skull thickness, density, and morphology further alter in situ pressure fields, leading to variability in effective dose delivery. Although advances in acoustic modeling, neuronavigation, and phased-array technologies aim to mitigate these issues, they are not yet consistently implemented or widely accessible. Moreover, no predictive framework currently exists to determine which targets or patients are most likely to respond, underscoring the need for integrative approaches combining transcriptomics, electrophysiology, imaging, and individualized acoustic simulations.

Third, safety, regulatory pathways, and clinical readiness remain incompletely established. While low-intensity TUS appears well tolerated in early human studies, the relationship between stimulation intensity, efficacy, and risk is not linear, and thresholds for adverse effects such as thermal accumulation, cavitation, or subtle tissue changes remain poorly defined. Long-term safety data and the effects of repeated exposure are limited. In parallel, regulatory frameworks are still evolving, and there is no consensus on standardized protocols, reporting guidelines, or clinically approved dosing strategies comparable to those established for DBS or other neuromodulation modalities. Although efforts such as the ITRUSST consensus provide important guidance on safety and reporting, these remain recommendation-based rather than prescriptive, clinically validated standards. Together, these factors position TUS as an emerging, early-stage technology rather than a clinically mature therapy.

In summary, TUS is a promising, patient-friendly neuromodulation approach with a unique capacity for non-invasive deep brain targeting. However, key translational barriers including parameter standardization, inter-individual variability, mechanistic uncertainty, and regulatory development must be addressed before widespread clinical adoption. Progress will depend on harmonized reporting standards, large-scale multi-site sham-controlled trials, real-time imaging, improved targeting and aberration correction, and the integration of biomarker- and AI-guided closed-loop frameworks to validate efficacy and enable personalization. If these challenges are overcome, TUS may evolve into a clinically viable, non-invasive complement to invasive neuromodulation techniques within the next decade.

## 4 Overall Conclusions

DBS and TUS are advancing along complementary trajectories toward the shared goal of more precise, circuit-based, biomarker-guided neuromodulation therapies. In DBS, the fusion of connectomic targeting (to determine where to stimulate based on individualized network maps) with adaptive stimulation (to determine when to stimulate based on real-time neural signals) defines the next generation of precision neuromodulation. This approach moves DBS beyond focal symptom management into an era of intelligent, closed-loop therapy that continuously interprets brain-state biomarkers (such as pathological oscillations) to optimize stimulation delivery. TUS is following a parallel trajectory: emerging protocols are beginning to incorporate real-time imaging guidance, acoustic feedback control, and even AI algorithms to tailor sonication to each patient’s brain dynamics.

Although DBS and TUS differ in both their technical foundations (electrical pulses versus acoustic waves) and their current stage of clinical translation (with DBS representing an established and continually refined modality and TUS still in early experimental phases) their underlying principles are rapidly converging. Ultimately, the field envisions delivering circuit-specific stimulation through the least invasive yet most effective means possible, combining the proven network engagement of DBS with the non-invasive precision of ultrasound. Both modalities increasingly rely on individualized targeting and neural biomarkers to guide stimulation in space and time. While DBS established the blueprint for circuit-based therapy, TUS is extending that blueprint into a non-invasive domain. This convergence between invasive and non-invasive strategies defines the next frontier in neuromodulation, as emerging therapies strive to deliver personalized, high-efficacy treatments while minimizing risk and invasiveness.

## Funding

This study received no external funding.

## Declaration of competing interest

The authors declare that they have no known competing financial interests or personal relationships that could have appeared to influence the work reported in this paper.
